# Vitamin D deficiency in early implant failure: two case reports

**DOI:** 10.1186/s40729-016-0056-0

**Published:** 2016-11-25

**Authors:** Tobias Fretwurst, Sebastian Grunert, Johan P. Woelber, Katja Nelson, Wiebke Semper-Hogg

**Affiliations:** 1 Department of Oral- and Craniomaxillofacial Surgery, Center for Dental Medicine, University Medical Center Freiburg, Hugstetter Straße 55, Freiburg, D-79106 Germany; 2 Department of Periodontics and Oral Medicine, University of Michigan School of Dentistry, Ann Arbor, USA; 3 Department of Operative Dentistry and Periodontology, Center for Dental Medicine, University Medical Center Freiburg, Hugstetter Straße 55, Freiburg, D-79106 Germany

**Keywords:** Vitamin D deficiency, Cholecalciferol, Early implant loss, Early implant failure, Bone grafting, Osseointegration, Osteoimmunology

## Abstract

An association between vitamin D deficiency and early dental implant failure is not properly verified, but its role in osteoimmunology is discussed. This article illustrates two case reports with vitamin D deficiency and early implant failure. Prior to implant placement, the first patient received crestal bone grafting with autologous material. Both patients received dental implants from different manufacturers in the molar region of the mandible. In the case of bone grafting in the first patient, all implants were placed in a two-stage procedure. All implants had to be removed within 15 days after implant placement. Vitamin D serum levels were measured: Both patients showed a vitamin D deficiency (serum vitamin D level <20 μg/l). After vitamin D supplementation, implant placement was successful in both patients. Prospective, randomized clinical trials must follow to affirm the relationship between vitamin D deficiency, osteoimmunology, and early implant failure.

## Background

Long-term stable osseointegrated implants are the primary goal in dental implantology. Although dental implants have proven clinical reliable in the long term, the failure of implants at a very early stage of osseointegration has been described [1, 2]. The pursuit to identify the mechanisms leading to early implant failure is ongoing to date and include the following: tobacco usage, diabetes, wear particle release and foreign body reaction, local bone necrosis due to heat generation during bone preparation or implant placement [2–9]. Systematic reviews demonstrated that an antibiotic regimen before dental implant placement subtly reduces the early implant infection and consequently implant failure [10, 11]. In orthopedics, the risk to develop a periprosthetic joint infection has been associated with a low vitamin D level [12]. A relationship between bone metabolism, vitamin D, and early implant failure in human has not been proven to date. Vitamin D induces bone formation around implants in rodents [13–17]. Besides the classical function, findings of the last decades indicate vitamin D as an important immune regulator targeting both, the innate and adaptive immune response, since all cells of the immune system express vitamin d receptor (VDR) [18–20].

The definition of vitamin D serum level is discussed in the literature [21–25]. Currently, vitamin D insufficiency is defined as serum level ranges between 21 and 29 μg/l, a serum level below <20 μg/l as vitamin D deficiency (severe deficiency <10 μg/l) [22, 25, 26]. The prevalence of vitamin D deficiency in Europe varies widely with ranges between 2 to 30% in adults and up to 80% in elderly patients [27]. Malnutrition, insufficient sun exposure, premature and dysmature birth, pigmented skin, obesity and advanced age are known as factors for a vitamin D deficiency [28].

A current review article demands the investigation of vitamin D deficiency in the context of dental implant failures [16]. To address this demand, the present case reports raised the question if vitamin D deficiency influences implant survival in the early stages of healing. This article illustrates two apparently healthy patients with vitamin D deficiency and early implant failure and demonstrates that implant placement was successful after vitamin D supplementation.

## Case presentation

### Patients and surgical procedure

Patients treated consecutively in one center (Department of Oral- and Craniomaxillofacial Surgery, University Medical Center Freiburg). None of the patients showed systemic disease. Both patients did not take regular medication and were negative for alcohol, nicotine, and drug use. Bothe male patients (48 and 51 years of age) were not immunosuppressed, irradiated or received chemotherapy. Written informed consent was obtained from the patient for publication of this case report and accompanying images. An ethic committee approval and consent was not necessary. This case report was performed in accordance with the Helsinki Declaration of 1964, as revised in 2013. Both patients lost their teeth several years before implant placement. The reason for extraction was not evaluable since both patients were referred for grafting and implant placement.

Surgery for bone augmentation and implant placement was performed under local anesthesia with Ultracain forte (Sanofi Aventis, Frankfurt, Germany) following a standard operation protocol. In the augmentation procedure, a crestal incision in the attached gingiva of the edentulous alveolar crest with one vertical releasing incision was used, after mobilization of the mucoperiosteal flap an autologous retromolar bone block was fixed on the occlusal part of the alveolar ridge. The bone block was secured with a microscrew (Modus 1.5, Medartis, Umkirch, Germany) and covered with a resorbable membrane (BioGide, Geistlich AG, Switzerland) as described previously [29]. The passively mobilized mucosa was closed with a running suture and secured with interrupted sutures (5-0 Monocryl, Ethicon, Norderstedt, Germany).

All implants were inserted with the same recommended torque of <35 Ncm. A standard oral antibiotic regimen was not applied after implant placement.

Both patients were clinically evaluated after one, ten and thirty days. All bone grafting procedures and implant placements were performed by two experienced surgeons (KN and SG). Oral radiographic examinations (orthopantomograms) were performed before and one day after the surgical procedure.

#### Patient

**Fig. 1:**
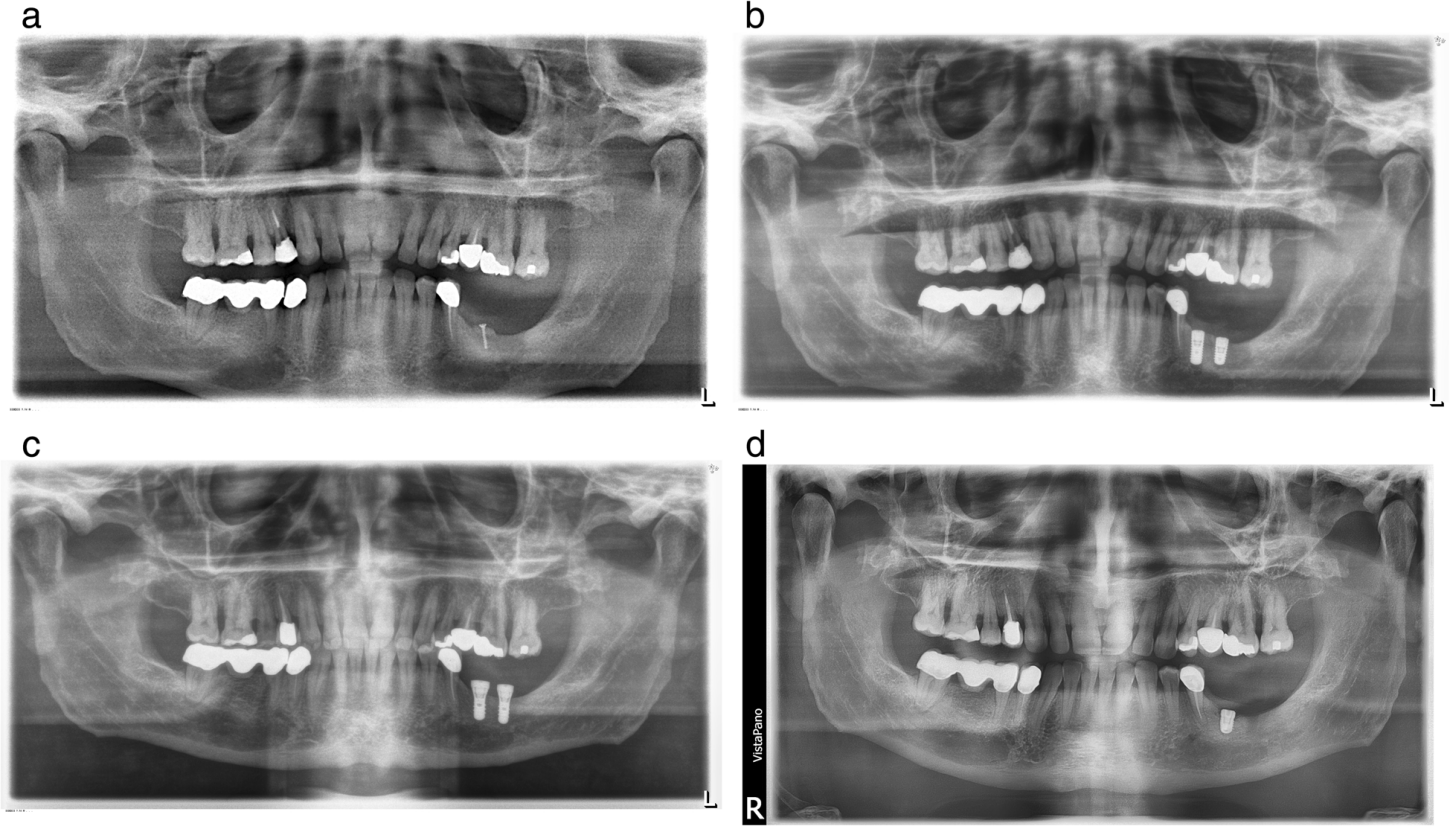
**a** Patient 1. Post grafting orthopantomogram. The bone block was secured with a single microscrew. **b** Patient 1. The radiograph demonstrates veritable inserted Straumann bone level implants after the first implant placement (1 day after implant placement). A peri-implant osteolysis is not visible. **c** Patient 1. Postoperative orthopantomogram (1 day after implant placement) after second implant placement of Straumann tissue level implants 6 months later. A peri-implant osteolysis is not visible. **d** Patient 1. Postoperative orthopantomogram after third implant placement (Conelog ScrewLine implant)

The medical history of this 48-year-old male patient showed a high blood pressure; otherwise, the patient was healthy. A successfully completed periodontal therapy was done before implant therapy. The patient demonstrated stable marginal bone levels. Autologous retromolar bone grafting using local anesthesia was performed in the left mandible (see Fig. 1a). This patient received a postoperative oral antibiotic therapy with amoxicillin 750 mg for 3 days. Three months after grafting two implants (Straumann, bone level) were placed in regions 36 and 37 (implant characteristic, see Table 1). During implant placement, cortical bone profiling was performed as recommended by the manufacturer guidelines. The bone graft appeared fully revascularized and incorporated. One day postoperatively the patient reported to have pain. The radiograph performed on that day demonstrated veritable inserted Straumann bone level implants. A peri-implant osteolysis was not visible (Fig. 1b). After 3 days of continuous severe pain, both implants were removed. At the time point of explantation, the local soft tissue showed no signs of inflammation. During surgical removal of the implants, an extensive osteolysis surrounding the implants was found and a thorough debridement of the area with a complete wound closure was performed. A second implant surgery was scheduled after 6 months with placement of two implants (Straumann, tissue level) in regions 36 and 37 (Fig. 1c). At reentry, the mandible appeared well vascularized; no granulation tissue was detectable. The drilling protocol was performed as recommended by the manufacturer including a cortical bone profiling before implant placement. Again after 3 days, both implants had to be removed because of continuous severe pain in the absence of any sign of soft tissue inflammation, swelling, or abscess formation.

**Table 1:** Implant characteristics—insertion region/explantation

Patient	Implant region (FDI)	Implant parameters	Dimensions of implant Diameter [mm]/length [mm]	Explantation [Days after placement]
1	36 37	First placement Straumann RN SLactive® (TiZr)	First placement Ø: 4.1; L: 10 Ø: 4.1; L: 8	3
	36 37	Second placement Straumann Tissue level (TiZr)	Second placement Ø :4.1, L: 8 Ø :4.1, L: 8	3
	36	Third placement Conelog ScrewLine (TiGr.4)	Third placement Ø :4.3 L: 7	Successful
2	36 37	First placement Straumann RN SLactive® TiZr	First placement Ø: 4.1; L: 12 Ø: 4.1; L: 10	15
	36 37	Second placement Straumann RN SLactive® TiZr	Second placement Ø: 4.1; L: 10 Ø: 4.1; L: 10	Successful

At this time point, a screening of the relevant parameters for bone metabolism was performed and a vitamin D deficiency (vitamin D serum level 11 μg/l) was diagnosed. After vitamin D supplementation and a healing period of 6 months, a third surgical intervention was planned and one implant (Conelog ScrewLine) was inserted in region 36 (see Table 1 and Fig. 1d). During implant placement, the former explantation site appeared clinically fully re-ossified. The patient received an intraoperative intravenous single-dose antibiotic therapy with Isocillin 1.2 mega. At this time point, the patient demonstrated a vitamin D level of 46 μg/l. Second-stage surgery was successfully performed and the prosthetic restoration initiated. For all implants, a primary stability had been achieved.

#### Patient

**Fig. 2:**
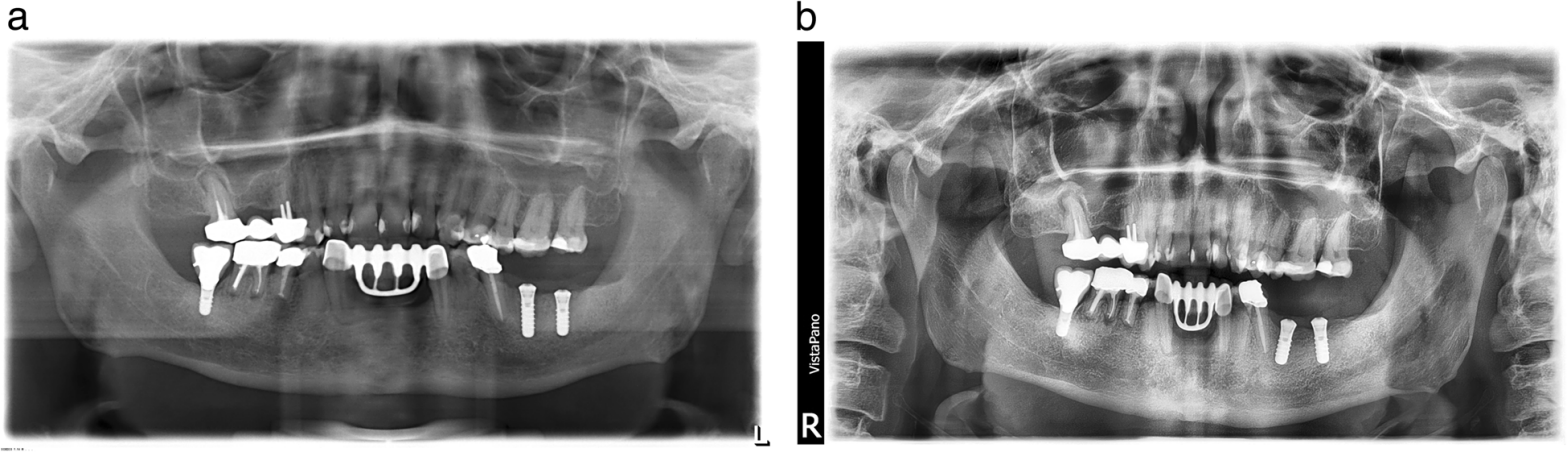
**a** Patient 2. Postoperative orthopantomogram one day after implant placement. **b** Patient 2. Postoperative orthopantomogram after second Implant placement

In this 51-year-old male patient, no grafting procedure was performed as vertical and horizontal alveolar ridge dimension was adequate for implant placement. The implant placement in regions 36 and 37 was performed as guided surgery (Fig. 2a, Table 1). The implant placement was uneventful and the bone appeared clinically healthy. A cortical bone profiling was performed during implant placement. One day postoperatively, the patient reported of a discomfort in the area of the operation but not severe pain. On day 7 postoperatively during a routine examination, the patient complained of continuous increasing severe pain since the operation. A medicinal pain therapy (ibuprofen 600 mg) was ineffective so that an explantation of both implants was performed at day 15. The serum vitamin D level showed an incipient deficiency (serum vitamin D level 20 μg/l). After vitamin D supplementation, a second implant placement in region 37 was performed successfully 4 months later. In region 36, a bony defect filled with granulation tissue was discovered and a debridement performed. Implant placement in region 36 was performed 8 weeks after the debridement (6 months after the first implant placement) (see Fig. 2b). The follow-up of both implants was uneventful and the prosthetic restoration performed. For all implants, a primary stability has been achieved. In both patients, appropriate torque was achieved using the system specific torque meter. After detailed vitamin D anamnesis malnutrition and insufficient sun exposure could be excluded as cause for vitamin D deficiency in both patients.

### Discussion

This article demonstrated that implant placement was successful after vitamin D supplementation in patients with vitamin D deficiency and early failed implants. None of the patients showed systemic disease or did take regular medication, alcohol, nicotine, or drugs. The patients were not immunosuppressed, irradiated, or received chemotherapy. All implants were inserted with the same recommended torque of <35 Ncm. Bone overheat or compression due to implant preparation/placement or contamination of implants surface during the surgical procedure were excluded due to a sufficient irrigation and the surgical protocol. In the end, only common obvious deficit of both patients was vitamin D deficiency. The blood analysis was otherwise unsuspicious. However, in the first patient, implants were placed 3 months after vertical autogenous bone augmentation with no systemic antibiotic treatment while antibiotics were given during the third successful attempt several months after guided bone regeneration. Missing antibiotic treatment and the grafting procedures have to be considered as conceivable factors for the early implant failure in the present patients nonetheless [10, 11]. To date, a vitamin D screening before implant placement has not become standard in our clinic; but in patients with early implant failure, a vitamin D screening is initiated.

Local and systemic factors can affect the survival rate of dental implants [30–33]. The causes of early implant failure are not fully clarified and an association between vitamin D and dental implant osseointegration has not been investigated properly [8, 13–16]. Some recent animal studies in rodents demonstrated a relationship between vitamin D supplementation and an increased bone to implant contact/volume after implant placement [14, 34, 35]. Mengatto et al. demonstrated an impaired osseointegration in vitamin D-deficient rats [36]. Other authors cannot confirm an effect of vitamin D supplementation on bone formation around titanium implants in diabetic rats [37]. A combination of local or systemic calcium supplementation and vitamin D seems to influence bone regeneration in extraction sockets of the dog as it demonstrates significantly higher bone formation and bone density and significantly less vertical ridge reduction in contrast to sockets without supplementation [38, 39]. However, a present human study cannot confirm an effect due to vitamin D supplementation on bone formation or graft resorption after maxillary sinus augmentation [40]. Satue et al. found a positive influence of 7-dehydrocholesterol (7-DHC), the precursor of vitamin D, coated implants on osteoblast differentiation in vitro [41]. But whether vitamin D-coated dental implants have an effect of osseointegration in vivo is still unclarified [42].

In dental implantology, vitamin D has been investigated almost exclusively as influencing factor of the bone to implant contact and implant stability [17]. Vitamin D demonstrates several effects on bone metabolism: it upregulates the gene expression of osteocalcin, osteopontin, calbindin, and 24-hydroxylase, increases extracellular matrix protein formation by osteoblasts, and stimulates osteoclast activity [15, 43]. But beyond modulation of bone formation, vitamin D has an impact on the innate and adaptive immune response in the field of osteoimmunology and could therefore influence early implant healing [19, 44–49].

Bone necrosis during implant bed preparation or placement is considered as a reason for early implant failures [7, 8]. An additional vitamin D deficiency might disrupt the sensitive balance between the immune system and bone metabolism during implant healing due to direct or indirect alteration of osteoclast function. For instance, the removal of bone debris through osteoclasts could be hampered since vitamin D controls osteoclast precursor monocyte migration [50]. On the other hand, in vitro studies demonstrated that vitamin D inhibits dendritic cell maturation and function as well as T cell proliferation and influences B cell responses, inhibiting proliferation and plasma cell differentiation [51–54]. An altered cytokine release by immune cells caused by a low vitamin D level could lead to a dysregulation of osteoclast activation and differentiation via associated immunoreceptors in osteoclasts [55].

Nevertheless, the vitamin D deficiency prevalence in the European population indicates that a vitamin D deficiency is probably not a sole causative factor for early implant failure; otherwise, the early implant failure rate would be significantly higher. However, a synergistic effect with other factors is conceivable. Some authors stated that implant osseointegration is not simply a wound healing phenomenon but rather complex foreign body reaction with activation of the immune system [56]. Titanium and metal particle release is discussed as cause for implant failure as well as implant dentistry [5, 6, 9, 56, 57]. It is assumed that metal particles influence the macrophage or lymphocyte pathways and provoke a release of pro-inflammatory cytokines, leading to an increased osteoclastogenesis and decreased osteoblastogenesis and consequently to peri-implant bone loss [58]. This osteolytic effect could be enhanced by vitamin D deficiency, since Maier et al. demonstrated in an epidemiological study that vitamin D deficiency is associated with a higher risk to develop aseptic loosening around joint replacements caused by wear particles [59]. Vitamin D could also be essential for the antibacterial response, as monocyte-macrophage reaction is influenced by vitamin D [60]. Xu et al. demonstrated that vitamin D can inhibit *Porphyromonas gingivalis*-induced proinflammatory cytokine expression and improve the expression of anti-inflammatory cytokines in macrophages [53].

Interestingly, in the present investigation, the osseointegration of dental implants seems to be more influenced by the vitamin D level than the autologous graft incorporation as the graft incorporation was not compromised. The eventually minor effect of vitamin D on the incorporation of bone grafts appears to be addressed by the results of a current randomized, double-blind, and placebo-controlled clinical investigation with a high-dose vitamin D3 supplementation combined with calcium. The results could not demonstrate a statistically significant difference in the amount of bone formation or graft resorption after maxillary sinus augmentation compared to a placebo medication [40].

## Conclusions

To overcome the shortcomings of this case reports, prospective, multicenter, and controlled studies must follow to affirm a potential relationship between vitamin D deficiency, osteoimmunology, and the early failure of dental implants. Currently, a general recommendation for a standardized vitamin D screening in dental implantology cannot be stated due to lack of evidence.
